# Pac-Man like DNA cut-and-paste tool allows larger gene edits

**DOI:** 10.1038/s42003-020-01446-7

**Published:** 2020-11-20

**Authors:** Anam Akhtar

**Affiliations:** Communications Biology, https://www.nature.com/commsbio

## Abstract

The CRISPR-Cas toolbox allows genetic manipulation of cultured cells, plants and animals on the basis of simpler RNA-guided DNA recognition. It has provided breakthrough scientific opportunities to engineer desirable traits, cure genetic diseases and enable point-of-care diagnostics. A recent study by Joseph Bondy-Denomy and colleagues further equips this toolbox to cut larger chunks of DNA from a cell’s genome.

The enormous power of the CRISPR-Cas toolbox was commemorated this year with the Nobel Prize in Chemistry to two scientists Jennifer Doudna and Emmanuelle Charpentier, who first developed it in 2012. CRISPR-Cas systems are a diverse group of RNA-guided nucleases that give prokaryotes the ability to defend against viral invaders. Encountered with a phage, microbes can capture snippets of foreign genetic elements from viral DNA and incorporate it into their own genomic CRISPR array. Transcription of CRISPR arrays creates CRISPR RNAs (crRNAs) that bind to Cas nucleases and provide specificity by base-pairing with target nucleic acids.

Among the diverse naturally evolved CRISPR-Cas systems, gene editing applications have mostly focussed on single subunit Class 2 CRISPR systems (such as Cas9 and Cas12a). The widely used CRISPR-Cas9 ensemble is frequently referred to as molecular scissors which can rapidly and precisely excise a small bit of DNA at the targeted site. However, the leading Cas9 and Cas12a enzymes are limited in their ability to make large deletions.

Joseph Bondy-Denomy and colleagues in the UCSF Department of Microbiology and Immunology developed a new CRISPR tool where instead of Cas9, they used a Class 1 system whose signature enzyme is Cas3^1^. Cas3, a 3′–5′ single-strand DNA helicase-nuclease enzyme can, unlike Cas9 or Cas12a, degrade much longer stretches of DNA quickly and accurately. Here, they repurposed and optimised this Type 1-C CRISPR system from *P. aeruginosa* (PaeCas3c) for both endogenous and heterologous genome engineering in four microbial species. They found it to be capable of efficient genome-scale deletions currently not achievable using other methodologies. Using only a single crRNA with modified repeat sequences, they isolated deletions with variable sizes, one as large as 424 kb, suggesting that this Cascade-Cas3 machinery is capable of degrading entire phage genomes.

Running along the length of the DNA and chewing it up like a Pac-Man, the CRISPR-Cas3 tool offers boundless possibilities, including facilitating the interrogation and manipulation of large segments of repetitive and non-coding DNA with unknown functions, having a broad impact on genetic research.

## References

[1] Csörgő, B. et al. A compact Cascade–Cas3 system for targeted genome engineering. *Nature Methods*10.1038/s41592-020-00980-w (2020). 10.1038/s41592-020-00980-w PMC7611934 33077967

